# Consensus on Screening, Diagnosis, and Staging Tools for Prostate Cancer in Developing Countries: A Report From the First Prostate Cancer Consensus Conference for Developing Countries (PCCCDC)

**DOI:** 10.1200/GO.20.00527

**Published:** 2021-04-15

**Authors:** Arie Carneiro, Douglas Racy, Carlos Eduardo Bacchi, Katia Ramos Moreira Leite, Renee Zon Filippi, Igor Austin Fernandes Martins, Joao Victor Salvajoli, Rodrigo de Morais Hanriot, Ronaldo Hueb Baroni, Alvaro Sadek Sarkis, Antonio Carlos Lima Pompeo, Bruno Santos Benigno, Gustavo Cardoso Guimarães, Saad Aldousari, Aguinaldo Cesar Nardi, Alexandre Saad Feres Lima Pompeo, Amr Nowier, Archimedes Nardozza Jr, Ari Adamy Jr, Celso Heitor de Freitas Jr, Daher Cezar Chade, Danilo Armando Citarella Otero, Deusdedit Cortêz Vieira da Silva Neto, Eduardo Franco Carvalhal, Fernando Korkes, Robson Ferrigno

**Affiliations:** ^1^ Hospital Israelita Albert Einstein, Sao Paulo, Brazil; ^2^ Hospital Beneficiencia Portuguesa de São Paulo, São Paulo, Brazil; ^3^ Laboratorio Bacchi, São Paulo, Brazil; ^4^ Faculdade de Medicina USP, São Paulo, Brazil; ^5^ HCOR, São Paulo, Brazil; ^6^ Hospital Alemao Oswaldo Cruz, São Paulo, Brazil; ^7^ Faculdade de Medicina do ABC, Santo André, Brazil; ^8^ Kuwait University, Kuwait City, Kuwait; ^9^ Unimed Bauru, Bauru, Brazil; ^10^ Ain Shams University, Cairo, Egypt; ^11^ UNIFESP, São Paulo, Brazil; ^12^ Hospital Santa Cruz, Santa Cruz do Sul, Brazil; ^13^ Universidad del Rosario, Bogota, Colombia; ^14^ Faculdade de Medicina da Santa Casa, São Paulo, Brazil; ^15^ Hospital Moinhos de Vento, Porto Alegre, Brazil

## Abstract

**PURPOSE:**

To generate and present the survey results on critical issues relevant to screening, diagnosis, and staging tools for prostate cancer (PCa) focused on developing countries.

**METHODS:**

A total of 36 of 300 questions concern the main areas of interest of this paper: (1) screening, (2) diagnosis, and (3) staging for various risk levels of PCa in developing countries. A panel of 99 international multidisciplinary cancer experts voted on these questions to create recommendations for screening, diagnosing, and staging tools for PCa in areas of limited resources discussed in this manuscript.

**RESULTS:**

The panel voted publicly but anonymously on the predefined questions. Each question was deemed consensus if 75% or more of the full panel had selected a particular answer. These answers are based on panelist opinion not a literature review or meta-analysis. For questions that refer to an area of limited resources, the recommendations consider cost-effectiveness and the possible therapies with easier and greater access. Each question had five to seven relevant answers including two nonanswers. The results were tabulated in real time.

**CONCLUSION:**

The voting results and recommendations presented in this document can be used by physicians to support the screening, diagnosis, and staging of PCa in areas of limited resources. Individual clinical decision making should be supported by available data; however, as guidelines for screening, diagnosis, and staging of PCa in developing countries have not been developed, this document will serve as a point of reference when confronted with this disease.

## INTRODUCTION

The incidence of prostate cancer (PCa) has been increasing worldwide in recent years. GLOBOCAN data showed that PCa was the second most frequently diagnosed cancer and the fifth leading cause of cancer mortality among men worldwide in 2012. Data reveal that prostate-specific antigen (PSA) testing and disease incidence have risen significantly in developing and Asian countries and PCa has become one of the leading male cancers in many of these nations.^1^

**CONTEXT**

**Key Objective**
To generate a consensus on critical issues relevant to screening, diagnosis, and staging tools for prostate cancer (PCa) focused on developing countries.
**Knowledge Generated**
Most of the panel recommended a universal screening strategy for a target population of Black males 45 years or older with a familiar history of PCa and all males 50 years or older. Baseline prostate biopsy should ideally be performed by transrectal ultrasound–guided biopsy with ≥ 8 random cores (for a prostate volume of 30-40 mL) and 10-12 cores for high-volume prostates. The panel agreed that no method is indicated for staging low-risk PCa in patients who have already undergone a biopsy. Computed tomography of the thorax or chest x-ray, abdominal and pelvic computed tomography, and bone scan are the most indicated methods for staging.
**Relevance**
The voting results presented in this document can be used to support the screening, diagnosis, and staging of PCa in areas of limited resources lacking specific guidelines.

PCa is currently the second most common cause of death among men. It is a tumor of elderly men with a mean age at diagnosis of 72 years and accounts for about 15% of malignancies in men in developed countries and 4% in developing countries.^2,3^ Since 1985, a significant increase in the number of PCa-related deaths was observed in most European countries, even in countries or regions where PCa is not frequent.^4^ The Asian-Pacific region, which comprises 35% of the world’s male population, had approximately 14% (122,000) of all PCa diagnosed worldwide in 2008 (10 per 100,000 population), and there were about 42,000 deaths because of PCa (three per 100,000). In Brazil, 12,778 PCa deaths were reported and more than 60,000 new cases were diagnosed in 2012.^5^ However, the lack of population-based data across most of the countries in this region limits the ability of researchers to understand and report on the patterns and distribution of this important cancer.^6^

In Africa, although it is still quite difficult to accurately establish the burden of PCa because of poor cancer registration systems, reports show that African men disproportionately present with PCa compared with men from other parts of the world.^7^ In the GLOBOCAN 2012, PCa incidence and mortality rates in Africa were reported to be 23.2 and 17.0 per 100,000, respectively. In fact, the evidence shows that mortality rates from PCa are higher in Black African populations compared with other races.^7^

In Latin America, PCa is of particular concern because of its high prevalence in the region and its continuing upward trajectory. It is the leading cause of cancer-related deaths among Latin American males, and the disease and economic burden are set to rise in tandem with longevity and changes in lifestyle and diet. Risk factors are highest in Brazil, which has a more rapidly aging population and a greater proportion of males of African descent than in other countries in the region. The rates of incidence of PCa and deaths from this disease are expected to double in the region by 2030, according to The Pan-American Health Organization.^8^

Population screening with PSA and digital rectal examination (DRE) can detect early disease and offers the potential to decrease morbidity and cancer-specific mortality (SOURCE).^9,10^ As a result, PCa screening plays a key role in developing countries with potential benefits that transcend PCa management. During the routine visit for PCa screening, it is possible to review other clinically significant areas such as blood pressure and blood glucose and identify additional risk factors for other diseases such as cardiovascular disease. However, despite potential and expected better outcomes from PCa screening and early detection, benefits from PCa screening remained unproven before 2018.^1^ This meeting promoted the discussion to find common ground in those limited-resource settings, providing an alternative recommendation to support decision making in PCa for these areas.

## SCREENING

In 2012, the US Preventive Services Task Force (USPSTF) published a recommendation against systematic screening, creating confusion. A study presented at the 2015 Genitourinary Cancers Symposium sparked even more discussion on the topic by reinforcing the role of screening after showing a 3% per year increase in the diagnosis of intermediate- and high-risk tumors during the years after the USPSTF issued the recommendation.^9,10^

Currently, the Brazilian Society of Urology (SBU) recommends that 50-year-old men should seek a specialized professional for an individualized assessment and start annual screening with rectal examination and PSA.^11^ The USPSTF provides a grade C recommendation for periodic prostate screening for men 55-69 years and recommends against screening for men 70 years or older. Because PCa often grows slowly, men without symptoms of PCa whose life expectancy is < 10 years should not be offered testing since they are not likely to benefit. Table 1 summarizes the most important screening recommendations.

**TABLE 1:**
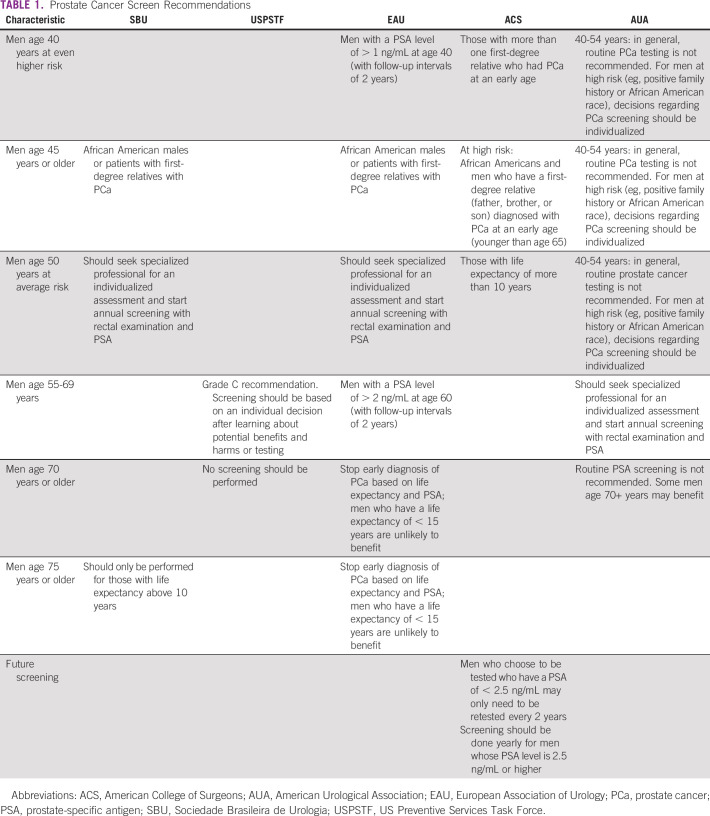
Prostate Cancer Screen Recommendations

Regarding counseling a specific healthy male patient looking for information on PCa screening, after presenting risks and benefits of screening, and looking for a shared decision, while the panel did not reach a consensus, most (60.98%) would advise in favor of regular PCa screening. However, some panel members (37.80%) would advise in favor of regular PCa screening only for selected cases.

When advising public health authorities in a country with limited resources regarding population screening for PCa, the panel was split. Half of the panel (47.56%) recommend an individualized decision, presenting risks and benefits of screening looking for a shared decision. The other half of the panel (45.12%) advise in favor of regular PCa screening, with well-structured and strict protocols for high-risk populations.

The panel reached consensus (78.05%) in recommending the adoption of a universal screening strategy for a target population of Black males 45 years or older with a familiar history of PCa and all males 50 years or older. However, in a country with limited resources, most of the panel (68.67%) recommended a universal screening strategy for the same target population. One fifth of the panel (19.28%) recommended that the target population should be 50 years old.

Although the panel reached consensus that the PSA and DRE tests must be used for prostate screening for both the general population (82.35%) and in countries with limited resources (82.93%), it differed somewhat in the frequency of screening. In general, the panel advises that when PCa screening is recommended, it should be applied yearly (46.99%) or depending on PSA levels (32.53%). However, when screening is recommended in countries with limited resources, the panel advises screening depending on PSA levels (39.51%), every 2 years (24.69%) or yearly (22.22%).

## DIAGNOSIS

Although PSA levels historically correlate with the presence of PCa, this test provides little information regarding disease location and extent. As PCa is diagnosed at progressively lower levels of serum PSA, clinicians have sought to identify better means of diagnosing, staging, and monitoring patients with the malignancy. In men with low-risk disease parameters, imaging provides little information regarding stage, and its use is infrequent. In high-risk patients, imaging confirms or rules out the presence of metastatic disease, but in most cases, the absence of disease on imaging does not change the choice of therapy or disease-related prognosis.^12^

Transrectal ultrasound (TRUS)–guided biopsy is considered the standard of care for the diagnosis of PCa in men presenting with elevated PSA levels or abnormal DRE. This systematic sextant extended biopsy approach samples 0.04% of the prostate volume and yields cancer detection rates of only up to 40%. Since TRUS biopsies are not targeted, they can lead to overdiagnosis of clinically indolent tumors, while missing clinically significant PCa foci.^13^

Technological advances in imaging have created a new role for various tests in the management of PCas. Advances in imaging evaluate the biology of the disease and, in doing so, allow more accurate detection of the location, extent, and aggressiveness of the malignancy.^12^ Magnetic resonance imaging (MRI) of the prostate may be used in many clinical scenarios, including primary screening, active surveillance, and in patients with a previous negative biopsy and rising PSA level.^14^ Technical advances in MRI in the last decade have made this method the preferred imaging modality for prostate anatomy and PCa risk assessment. As of 2018, the indications for MRI in the diagnosis and risk assessment of PCa have expanded from preoperative evaluation to the prebiopsy setting and for surveillance protocols.^13^

Recently, several advances in prostate imaging have made multiparametric MRI (MP-MRI) the preferred imaging modality for detecting areas suspicious for PCa and allowing for targeted biopsy sampling.^13^ The combination of morphologic and functional MRI information is accepted as the best imaging correlate for PCa diagnostics.^15^ Although many studies at 1.5T have shown a clear diagnostic benefit with an additional endorectal coil, the need for an endorectal coil at 3T is still controversial given the increased effort and patient discomfort.^13^ Research on MP-MRI and targeted biopsies is ongoing, providing more evidence on their use in the detection and risk stratification of PCa.^15^ No prospective high-impact data are available regarding prostate-specific membrane antigen (PSMA) positron emission tomography (PET) and/or MRI for assessment of therapeutic response in PCa. Despite the great potential of this modality and encouraging preliminary results, further trials are necessary to provide a better clinical understanding of PSMA expression behavior compared with various therapy modalities (mainly androgen deprivation treatment), to optimize molecular and functional response criteria, and to improve both PET and MRI quantitative algorithms.^16^

When asked how frequently to recommend image tests for all patients with PSA alterations and/or abnormalities in DRE before biopsy, as it can avoid unnecessary biopsy and/or guide a targeted biopsy, the panel reached consensus (79.07%) in voting to always recommend image tests for all these patients. However, when considering the same recommendation in an area of limited resources, only about one fifth (21.18%) of the panel voted to always make that recommendation, whereas most of the panel (69.41%) would make the recommendation just for selected cases (eg, re-biopsy).

Despite not reaching a consensus, most of the panel (70.73%) voted to use prostate MP-MRI (3T) as the imaging method that is indicated for the detection of PCa in patients with PSA alterations and/or abnormalities in DRE before biopsy. When considering an area with limited resources and with no prostate 3T MRI available, the panel split, with more (40.00%) selecting no imaging method and to proceed to biopsy, some (24.71%) selecting prostate mpMRI (1.5T without rectal coil), and others (22.35%) selecting prostate TR-US.

When all complementary investigations are available, the panel reached consensus in selecting prostate MRI (75.00%) as the strategy to be offered to avoid unnecessary biopsies for patients with a PSA level of 2-10 ng/mL. However, if no specific imaging or additional serum or urine-based markers are available, to avoid unnecessary biopsies for patients with more than one altered PSA (PSA, 2-10 ng/mL), with an upward trend, and no clinical symptoms of urinary tract infection, the panel split in recommending biopsy immediately (48.44%) and repeat PSA in 3 months (35.94%).

When asked how the baseline prostate biopsy should ideally be performed, a consensus was nearly reached by the panel (74.42%) in voting for fusion ultrasound-MRI–guided biopsy with ≥ 8 random cores (for a prostate volume of 30-40 mL) and 10-12 random cores for high-volume prostates and 2-3 target cores. However, almost one fifth of the panel (19.77%) voted for transrectal ultrasound–guided biopsy with ≥ 8 random cores (for a prostate volume of 30-40 mL) and 10-12 random cores for high-volume prostates.

When considering that MRI is not available, the panel reached a strong consensus (95.29%) in recommending that the baseline prostate biopsy should ideally be performed by transrectal ultrasound–guided biopsy with ≥ 8 random cores (for a prostate volume of 30-40 mL) and 10-12 cores for high-volume prostates.

If a second biopsy is to be performed, the panel split on the recommended technique to be used, with more than half (58.02%) selecting MRI plus fusion-guided biopsy using the validate software or cognitive approach and almost a third (30.86%) selecting MRI plus fusion-guided biopsy using the validate software obligatory.

When an MRI is not available, the panel again split in recommending an approach to perform a second biopsy with more than half (56.47%) voting for saturation biopsy with TRUS-guided biopsy with ≥ 20 cores and a little more than a quarter (27.06%) voting for transrectal ultrasound–guided biopsy with ≥ 8 cores (for a prostate volume of 30-40 mL) and 10-12 cores for high-volume prostates.

## STAGING TOOLS

When asked which imaging method is indicated for staging low-risk PCa in patients who have already undergone a biopsy, the panel split in their recommendation with more (46.75%) selecting MRI of the prostate only if not done before the biopsy and some (37.66%) recommending no imaging method. In circumstances where there is a certain unavailability of the imaging resources, the panel reached a consensus (82.93%) and agreed that no method is indicated for staging low-risk PCa in patients who have already undergone a biopsy.

For staging localized intermediate-risk PCa, most of the panel (62.79%) recommended a combination of bone scan and computed tomography (CT) scan of the abdomen and pelvis and chest x-ray or CT scan of the thorax and MRI of the prostate if not done before the biopsy, in patients who have undergone a biopsy, whereas some (24.42%) recommend MRI of the prostate only if not done before the biopsy. However, where there is a certain limitation in the availability of imaging resources, most of the panel (56.47%) recommended a combination of bone scan and chest x-ray or CT scan of the thorax and CT scan of the abdomen and pelvis as the best method indicated. No other option received above 13% of the panel’s vote.

In patients with high-risk PCa who have already undergone a biopsy, most of the panel (67.07%) recommend a combination of bone scan and CT scan of the abdomen and pelvis and chest x-ray or CT scan of the thorax and MRI of the prostate if not done before biopsy as the preferred imaging method, whereas some (21.95%) recommend PET and/or CT (PSMA, choline, or FACBC [fluciclovine]). Where there is a certain limitation in the availability of imaging resources, most of the panel (70.24%) recommend a combination of bone scan and chest x-ray or CT scan of the thorax and CT scan of the abdomen and pelvis, whereas one quarter (25.00%) recommend a combination of bone scan and CT scan of the abdomen and pelvis and chest x-ray or CT scan of the thorax and MRI of the prostate if not done before biopsy.

When considering which imaging method is indicated for castration-sensitive patient with biochemical recurrence after radical prostatectomy, the panel reached a strong consensus (91.03%) in selecting PET-CT with PSMA (or PET-MRI with PSMA) with or without pelvic MRI. However, for the same patient and circumstance, where there is a limitation in the availability of imaging resources, the panel split with most (62.34%) recommending CT of the thorax or chest x-ray, CT of the abdomen and pelvis (or pelvic MRI), and bone scan and some (22.08%) selecting pelvic MRI and bone scan.

For castration-sensitive patients with biochemical recurrence after radiotherapy, the panel reached a very strong consensus (98.51%) in recommending PET-CT with PSMA (or PET-MRI with PSMA) with or without pelvic MRI as the indicated imaging method. Yet, in circumstances where there is a limitation in the availability of imaging resources, for the same patient, while not reaching a consensus, most of the panel (70.42%) selected CT of the thorax or chest x-ray, CT of the abdomen and pelvis (or pelvic MRI), and bone scan as the indicated imaging method, with some (18.31%) recommending pelvic MRI and bone scan.

The panel reached a consensus (83.78%) in selecting PSMA PET and/or CT (or PSMA PET and/or MRI) with or without pelvic MRI as the indicated imaging method for a castration-resistant patient with biochemical recurrence after radical prostatectomy. In a limited-resource setting, for the same patient, the panel reached consensus (81.82%) in selecting CT of the thorax or chest x-ray, CT of the abdomen and pelvis (or pelvic MRI), and bone scan as the indicated imaging method.

For a castration-resistant patient with biochemical recurrence after curatively intended radiotherapy, the panel reached consensus (79.22%) in recommending PSMA PET-CT (or PSMA PET-MRI) with or without pelvic MRI as the indicated imaging method. In limited-resource settings, for the same patient, the panel reached consensus (93.59%) in recommending CT of the thorax or chest x-ray, CT of the abdomen and pelvis (or pelvic MRI), and bone scan as the indicated imaging method.

In the case of the patient with castration-naïve PCa and probable metastasis (M1), the panel split almost equally in its recommendation for the indicated imaging method, with almost half (47.14%) voting for thoracic CT or chest x-ray, abdominal and pelvic CT (or pelvic MRI), and bone scan and some (45.71%) voting for PET-PSMA. In the situation where not all the imaging methods are available, for this patient, the panel reached consensus (75.71%) in recommending thoracic CT or chest x-ray, abdominal and pelvic CT (or pelvic MRI), and bone scan as the indicated imaging method.

For the patient with metastatic castration-resistant prostate cancer, the panel split in its recommendation for the image studies indicated for staging, with more than half (55.42%) selecting thoracic CT or chest x-ray, abdominal and pelvic CT (or pelvic MRI), and bone scan and the remaining selecting (43.37%) PSMA PET. Additionally, for this patient in an area of limited resources, the panel reached consensus (82.05%) in recommending CT of the thorax or chest x-ray, abdominal and pelvic CT (or pelvic MRI), and bone scan.

In conclusion, given the high incidence of PCa globally, improved population-based cancer registries are critical for understanding the true burden of the disease in areas of limited resources to support the prioritization of PCa country-wide policies. PCa screening in high-risk populations can detect early disease and offer the potential to decrease morbidity and mortality and should be recommended following international guidelines. In addition, clinicians must be aware of the impact of a PCa diagnosis in a patient's quality of life and be able to accurately communicate the available treatment options and discuss active surveillance. Although modern technologies are helping advance the screening and diagnosis of PCa, they are currently not available in areas of limited resources and efforts should be made to ensure that the most cost-effective technologies are accessible to all patients.
